# Air Microbiome and Pollution: Composition and Potential Effects on Human Health, Including SARS Coronavirus Infection

**DOI:** 10.1155/2020/1646943

**Published:** 2020-05-28

**Authors:** Karin Moelling, Felix Broecker

**Affiliations:** ^1^Institute of Medical Microbiology, University of Zurich, Zurich, Switzerland; ^2^Max Planck Institute for Molecular Genetics, Berlin, Germany; ^3^Department of Microbiology, Icahn School of Medicine at Mount Sinai, New York, NY, USA

## Abstract

Polluted air poses a significant threat to human health. Exposure to particulate matter (PM) and harmful gases contributes to cardiovascular and respiratory diseases, including allergies and obstructive lung disease. Air pollution may also be linked to cancer and reduced life expectancy. Uptake of PM has been shown to cause pathological changes in the intestinal microbiota in mice and humans. Less is known about the effects of pollution-associated microbiota on human health. Several recent studies described the microbiomes of urban and rural air samples, of the stratosphere and sand particles, which can be transported over long distances, as well as the air of indoor environments. Here, we summarize the current knowledge on airborne bacterial, viral, and fungal communities and discuss their potential consequences on human health. The current data suggest that bacterial pathogens are typically too sparse and short-lived in air to pose a significant risk for infecting healthy people. However, airborne fungal spores may exacerbate allergies and asthma. Little information is available on viruses including phages, and future studies are likely to detect known and novel viruses with a yet unknown impact on human health. Furthermore, varying experimental protocols have been employed in the recent microbiome and virome studies. Therefore, standardized methodologies will be required to allow for better comparisons between studies. Air pollution has been linked to more severe outcomes of SARS (severe acute respiratory syndrome) coronavirus (SARS-CoV) infections. This may have contributed to severe SARS-CoV-2 outbreaks, especially those in China, Northern Italy, Iran, and New York City.

## 1. Introduction

### 1.1. Definition, Guidelines, and Sources of Air Pollution

Air quality is commonly described by the concentrations of PM (ranging in diameter from 10 *μ*m or smaller (PM_10_) to below 0.1 *μ*m (PM_0.1_)) and of the gaseous pollutants, ozone (O_3_), nitrogen dioxide (NO_2_), and sulfur dioxide (SO_2_) [1–5]. Current recommendations for PM_2.5_ and PM_10_ by the World Health Organization (WHO) are 10 and 20 *μ*g/m^3^ on average per year, respectively, while upper limits for NO_2_, O_3_, and SO_2_ are 40, 100 (at ground levels), and 20 *μ*g/m^3^, respectively (Table 1). Other agencies including the European Union (EU), the Chinese Ministry of Environmental Protection, and the United States Environmental Protection Agency (EPA) also publish guidelines (Table 1). Alarmingly, approximately 92% of the world's population lives in regions where WHO guidelines are not met [6].

**Figure 1:**
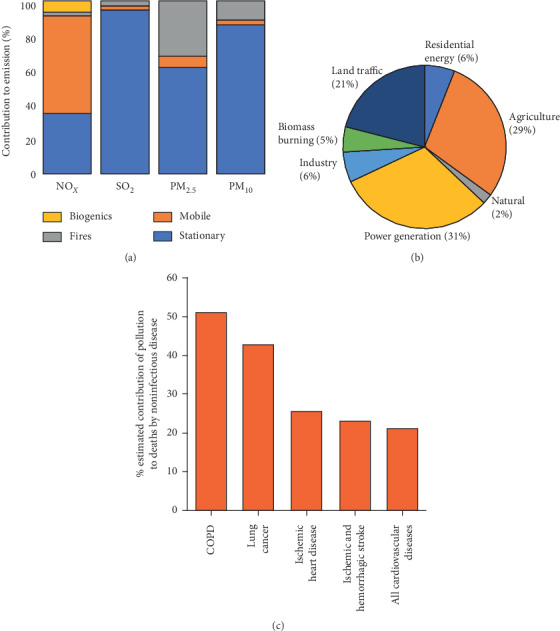
Origins of air pollution and contribution to deaths. (a) Relative contribution of different sources to the emission of NO_*X*_, SO_2_, PM_2.5_, and PM_10_ in the US for the year 2014 [7]. (b) Relative contribution of outdoor air pollution sources to premature death in the US for the year 2010 [9]. (c) Estimated contributions of global air pollution risk factors to deaths caused by noncommunicable disease for the years 1990–2015 [10].

Major emitters of air pollution are fossil fuel power plants, industry, agriculture, mobile sources (cars and other vehicles), wildfires, and natural processes occurring in various ecosystems [7–9]. In the US, mobile sources are the major emitters of nitrogen oxides (NO_*X*_), while stationary sources contribute mainly to emissions of SO_2_, PM_2.5_, and PM_10_ (Figure 1(a)) [7]. PM_10_ consists of particles produced by diesel fuel combustion, burning of wood, or biomass. PM_2.5_ emissions can result directly from exhaust of cars or other combustion processes and comprise soot particles. In addition, gases such as NO_*X*_, sulfur oxides (SO_*X*_), or ammonia (NH_3_) can form PM_2.5_, termed secondary fine dust, in a gas-to-particle conversion process [8]. Agriculture is the leading source of PM_2.5_ secondary fine dust in many parts of the world including Europe, which partially originates from NH_3_ of fertilizers (Figure 1(b)) [9].

## 2. Pollution-Related Diseases

The major health problems described for exposure to air pollution are likely caused by PM and mainly affect the airways and the cardiovascular system [10–16]. Both PM_10_ and PM_2.5_ can cause eye irritations, allergies, and rhinitis [10–12]. Globally, air pollution contributes to most deaths by chronic obstructive pulmonary disease (COPD) and to large proportions of fatalities caused by lung cancer, ischaemic heart disease, stroke, and cardiovascular diseases (Figure 1(c)). PM can also carry heavy metals that are associated with cancer and other diseases [17, 18]. The “Beijing Cough” is caused by polluting particles from inhaled smog [19]. A recent study described a correlation between PM_10_ and hospital admissions for respiratory diseases [20].

PM_2.5_ can reach deeper into the lung tissue than larger particles [21]. Long-time exposure has been correlated with increased risks of chronic diseases, including COPD that can develop into lung cancer (Table 2) [10–12, 19]. Cardiovascular diseases linked to PM_2.5_ include ischaemic heart disease and stroke [22].

Emerging evidence suggests causal associations between PM_2.5_ and type 2 diabetes, decreased cognitive functions, attention-deficit/hyperactivity disorder, autism, and neurodegenerative diseases [10]. PM_2.5_ may also be linked to premature birth, low birth weight, and sudden infant death syndrome [23]. However, these potential effects of air pollution remain to be better established and quantified.

Ultrafine nanoparticles (PM_1_ and PM_0.1_) can penetrate the skin, blood vessels, and the lymphatic system and are thereby distributed throughout the body; they can also act intracellularly [24]. Short-term exposure has been implicated in exacerbation of the abovementioned diseases, but long-term consequences are largely unknown [25]. However, nanoparticles have been found to induce oxidative stress, which is associated with neurodegenerative disorders, cancer, chronic fatigue syndrome, and cardiovascular and gastrointestinal diseases [26, 27]. Moreover, exposure to ultrafine nanoparticles has been linked to cardiovascular diseases in a recent cohort study [25].

Worldwide, about 3.3 million people die prematurely from outdoor air pollution each year [9], and additional about 3.8 million due to household pollution, mostly in developing countries where cooking with open fires is common practice [28]. Of these people, 27% die from pneumonia, 20% from COPD, 8% from lung cancer, and 45% due to cardiovascular diseases [10]. In Western countries, life expectancy is estimated to be reduced by an average of 8.3 months due to exposure to PM_2.5_ [29].

Globally, the effect of air pollution on life expectancy is estimated to be more than twice as strong as the effects of water, soil, and occupational pollution combined [10]. The estimated 9 million premature deaths annually due to indoor and outdoor air pollution exceed those estimated for smoking (about 7 million) and major infectious diseases (AIDS, tuberculosis, and malaria combined account for about 3 million premature deaths) [10].

### 2.1. Air Microbiome in Large Cities

Recently, the journal *BMC Infectious Diseases* published a special issue on the airborne microbiome, emphasizing on the spread of pathogens via human breath [30]. Here, we focus on pathogens potentially transported on the surface of airborne PM. The yet-unanswered question is whether and to what extent microbes carried by these particles contribute to diseases.

**Figure 2:**
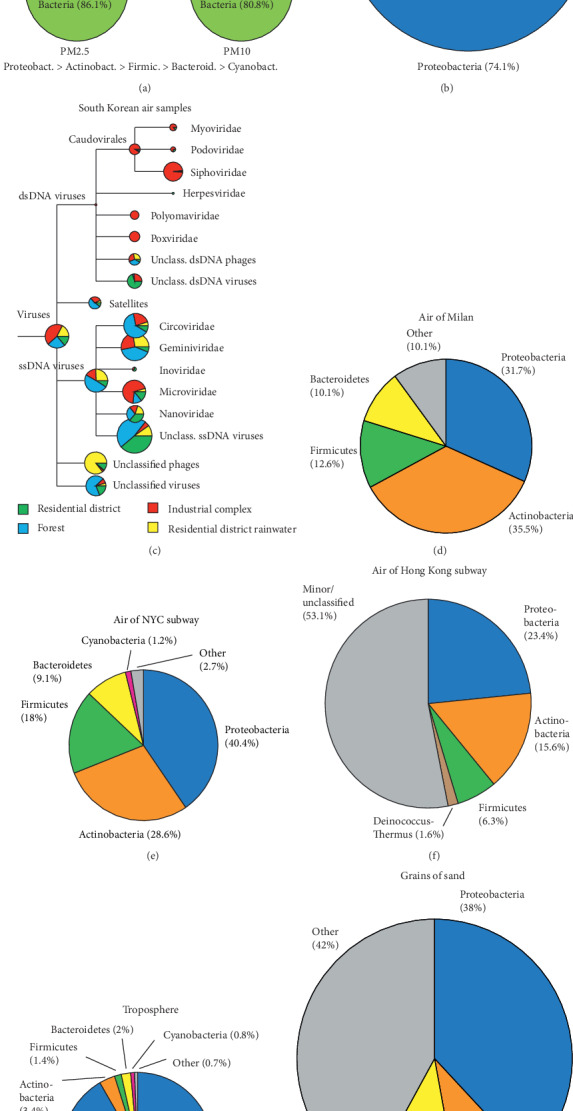
Compositions of air microbial communities in different locations. (a) Composition of the air microbes during a smog event in Beijing [31]. (b) Composition of the air bacterial communities during winter in the city of Urumqi [32]. (c) Virome of the air samples around Seoul, South Korea [33]. (d) Bacterial communities of air samples of the city of Milan during spring [34]. Bacterial communities of air samples obtained in the subway systems of New York City [35] (e) and Hong Kong [37] (f). Bacterial communities observed for the troposphere [38] (g) and on sand grains [39] (h).

The airborne microbiota of several cities have been characterized—Beijing [31] and Urumqi [32] in China, Seoul in South Korea [33], and Milan in Italy [34]. Additional studies investigated the subway systems of New York City, USA [35], Oslo, Norway [36], and Hong Kong [37]. In these studies, air filters were used to collect PM; microbial nucleic acids were isolated, processed, and sequenced. However, experimental conditions varied which limits direct comparison of the results. In Beijing, PM_2.5_ and PM_10_ levels of a severe smog event were analyzed over seven days and the extracted DNA was sequenced on an Illumina HiSeq 2000 sequencing system to determine microbial compositions [31]. Sequencing library preparation included a PCR step since amounts of DNA extracted from the air samples were too small for direct sequencing, and generated reads were aligned to nonredundant NCBI complete genomes for taxonomic assignment [31]. The detected microbes included bacteria (86.1% and 80.8% of reads in PM_2.5_ and PM_10_, respectively), eukaryotes (13% and 18.3%), 0.8% archaea, and 0.1% viruses in both samples (Figure 2(a)). The most abundant bacteria belonged to the Proteobacteria phylum, followed by Actinobacteria, Firmicutes, Bacteroidetes, and Cyanobacteria. Most inhalable microorganisms were soil-associated and nonpathogenic. However, microbes known to cause allergies and respiratory diseases were detected, including the bacterium *Streptococcus pneumoniae*, the fungus *Aspergillus fumigatus* that can cause asthma and respiratory aspergillosis [40], and human adenovirus C that causes respiratory, gastrointestinal, and urinary tract infections [41]. RNA viruses such as influenza, coronaviruses, or rhinoviruses were undetectable by the employed method. The authors concluded that there was likely no risk for contracting infectious diseases from pollutant-associated microbes, but they recommended fixing soil by vegetation to reduce the amount of airborne microbes originating from fecal and terrestrial sources, including potential allergens [31]. In this context, it is noteworthy that in 2018, China announced to promote revegetation and to increase forestation levels from about 22% in 2016 to 30% by 2050 to tackle air pollution [42].

Like in the air of Beijing, Proteobacteria and Actinobacteria were abundantly detected in the air of the city of Urumqi in northwest China (Figure 2(b)) [32]. This study also used DNA from filtered PM as the starting material, but taxonomic assignment was based on PCR-amplified 16S rRNA genes (prokaryotes) and 18S rRNA genes (eukaryotes) [32]. Several bacteria that may cause diseases in immunocompromised individuals but are typically harmless to the healthy population, such as *Acinetobacter*, *Delftia*, *Serratia*, and *Chryseobacterium* were detected. Some of the detected fungal spores are associated with allergies [43], such as Ascomycota, Basidiomycota, and Zygomycosis. Beijing is known for the “Beijing” cough, which affects many inhabitants independent of their age [19]. This condition may pose an increased risk for other lung diseases such as infection by SARS-CoV-2. Indeed, exposure to smog has been linked to an increased incidence of respiratory infections [44] and air pollution (such as PM and NO_2_) correlates with increased severity of diseases caused by infections with coronaviruses such as SARS-CoV-1 and SARS-CoV-2 [45–49]. SARS-CoV-2 may also be spread more efficiently in polluted air by attaching to PM [50].

A virome study from Seoul identified DNA viruses at different locations, industrial, residential, and a forest (Figure 2(c)) [33]. After removal of particles larger than 0.2 *μ*m by filtration, samples were concentrated by tangential flow filtration and virus particles were purified by CsCl density centrifugation. DNA was extracted and, without PCR amplification, subjected to 454 pyrosequencing. Reads were assigned to viral sequences using the CAMERA databases and were taxonomically assigned with Megan [33]. The study was not designed to detect any RNA viruses. The authors identified predominantly plant-infecting single-stranded DNA (ssDNA) viruses of the Gemini-, Nano*-* and Circoviridae families. Nanoviridae are aphid-transmitted plant viruses with circular ssDNA segments [51]. Circoviridae also have circular genomes and infect plants, birds, pigs, fish, and insects [52]. Geminiviridae consist of two capsids, each containing a circular ssDNA of opposite polarities [53]; some members can significantly damage crops [54]. In addition, Microviridae, ssDNA phages infecting *Enterobacteria*, were identified. The authors also detected Caudovirales, tailed phages with double-stranded DNA genomes. Microviridae and Caudovirales comprise the most abundant phage populations in the human intestinal tract [55] and have also been identified in marine environments [56, 57]. No human pathogenic viruses were detected. However, previously unknown ssDNA viruses were identified. Further studies will be necessary to address potential risks of the airborne virome on human health and on crop productivity.

A study in Milan, Northern Italy, evaluated forty air samples from ten days of sample collection during different seasons for bacterial and fungal communities (Figure 2(d)) [34]. The study relied on extracted bacterial DNA and PCR-amplified 16S rRNA genes that were sequenced on an Illumina Genome Analyzer IIx; taxonomic assignment was carried out with the RDP Bayesian Classifier [34]. Around 10^4^, mainly soil and plant-associated, bacteria per cubic meter of air were detected, with Actinobacteria and Proteobacteria dominating [34]. Significant seasonal and temperature-dependent variations were observed, for instance, with more Actinobacteria on colder days. The authors did not address whether potentially pathogenic or allergy-inducing species were detected.

The air of the New York City subway system was found to contain microorganisms mainly originating from outdoor air with a minor proportion from human skin [35]. Here, DNA extracted from filtered air samples was subjected to PCR to amplify 16S and 18S rRNA genes, the amplicons were subsequently sequenced by 454 pyrosequencing, and taxonomic assignment was achieved using the SILVA database [35]. On average, samples contained 40.4% Proteobacteria, 28.6% Actinobacteria, 18% Firmicutes, 9.1% Bacteroidetes, 1.2% Cyanobacteria, and a complex mixture of fungal spores (Figure 2(e)). Surprisingly, no known human pathogens were detected, but some of the detected fungi may cause allergies. The severity of the outbreak of SARS-CoV-2 in New York City may have been partly due to the high population density, high mobility, pollution, and also preexisting conditions such as obesity, which affects about 40% of the US population and may be a factor contributing to more severe outcomes of COVID-19 [48, 58].

In Oslo, aerosols were found to contain bacterial populations comparable to those of New York, with 37 different genera in total, some of them of skin origin [36]. Concentrations were about 10-fold lower at night [36]. Similarly, the air of the Hong Kong subway system predominantly contained Proteobacteria and Actinobacteria (Figure 2(f)) [37]. This study relied on extracted DNA subjected to PCR amplification of 16S rRNA genes and Illumina MiSeq sequencing. Taxonomic assignment was achieved by aligning reads against the Greengenes rRNA gene sequence database using the UCLUST program [37]. As observed in the New York City subway, bacterial communities showed significant similarities with those of outdoor air samples, with some human skin-associated bacteria also being present. Again, known pathogenic bacteria were not detected in this study.

### 2.2. Rural Air Microbiome

Besides soil bacteria, the Beijing study identified fecal bacteria as a prominent component of air pollution, possibly originating from rural animal farms. In addition, human fecal bacteria from sewage are a possible origin [31]. A study of the air microbiome of the grain-growing region Vaud, Switzerland, found a strong correlation between aerosolized and grain dust-associated fungal communities [59]. The presence of allergenic and mycotoxigenic species in most samples suggests that these fungal species may contribute to work-related respiratory symptoms of grain workers who, however, are exposed to much higher concentrations than the general population [59]. A study comparing rural and urban areas of the US found that urbanization leads to homogenization of the airborne microbiota, with urban communities exhibiting less geographic variability than rural areas [60]. The rural air microbiome was found to contain large numbers of fungi that are known triggers of allergies, including *Alternaria* and *Cladosporium* [60]. Further studies are needed to assess to what extent diseases may result from exposure to the rural air microbiome and how they correlate with concentrations and exposure times.

### 2.3. Microbiomes of the Troposphere, Dust, and Sand

There is evidence that microbes can be transported across very long distances and to high altitudes [60]. Bacteria represented on average 20% of particles between 0.25 and 1 *μ*m in diameter in either cloud-free or cloudy air obtained during the hurricanes Earl and Karl at 8–15 km altitude in the troposphere [38]. Numerous bacterial taxa were identified, including Acetobacteraceae, Burkholderiaceae, Streptomyces, and Pseudomonadaceae. Proteobacteria was the dominant phylum (Figure 2(g)). There were significant differences in microbial communities between samples from the two hurricanes. However, 17 bacterial and fungal species were common across all samples and may represent core members of the stratospheric microbiota [38]. Due to the poor resolution of the sequencing approach, the authors were unable to determine if any human pathogenic bacteria were present [38]. The vertical distribution of bacterial communities in the atmosphere above the Noto Peninsula, Japan, between 10- and 3,000-meter altitudes, has also been shown to vary substantially and mainly contained soil and marine bacteria [61]. The authors detected Bacilli and Proteobacteria, taxa that include known plant, animal, and human pathogens, which they speculated may be dispersed over large distances through high altitudes [61]. Whether these airborne pathogens can cause an infection after exposure to high altitude remains to be shown.

Sand grains can be transported over thousands of kilometers and transport bacteria, such that their populations may even be globally connected [62]. Sand grains of 200–600 *μ*m in diameter from a German shore were shown to bind 10^4^ to 10^5^ bacteria composed of 3,000 to 6,000 different species, mostly of soil and marine origin [39]. A core bacterial community was determined, with 50% of the bacteria present on all sand grains, and the other half varied. Proteobacteria was the dominant phylum, followed by Bacteroidetes and Actinobacteria (Figure 2(h)). The identified bacteria were not discussed as potentially harmful for people.

Dust from desert soil was shown to transport diverse assemblages of bacteria to the Mediterranean [63]. The dust microbiome of the Gobi Desert was found to contain large amounts of Alphaproteobacteria [64]. Soil bacteria were more abundant during dust storm events, while the relative abundance of bacteria of anthropogenic origin decreased [65]. Anthropogenic bacteria included those carrying antibiotic resistance genes, suggesting that the air microbiome may contribute to the spread of antibiotic resistance over long distances, whereby these genes may get diluted. No human health risks have been described [65]. A concern, however, is the presence of antibiotic resistance genes in the sewage of livestock production that can be transported by water or through air [66].

### 2.4. Indoor Air Microbiome

Indoor pollution has been analyzed systematically using household air [67–69]. Here, Western households must be distinguished from those in developing countries where open fires used for cooking contribute to pollution, a major health concern and cause of premature mortality [70]. In Western households, major sources of microorganisms are humans, pets, plants, plumbing, heating, ventilation/air conditioning, mold, and dust from outdoors [68]. People typically stay most of the day indoors, and the air microbiomes differ significantly between environments such as schools, offices, households, and transportation and even between different rooms of the same household [67–69]. One cubic meter of indoor air typically contains 10^5^ of both virus-like and bacteria-like particles, about a tenth of the concentrations found in outdoor air [68]. Fungal spores are less abundant and vary in numbers from around 80 up to 10^4^ colony-forming units. Humans emit around 10^7^ copies of bacterial and fungal genomes per hour [68]. Human stool can contain up to 10^9^ particles per gram of fecal-transmitted pathogens such as norovirus, *Shigella*, or *Salmonella* [71]. It should be noted that humans carry 10^12^ microorganisms on their skin and 4 × 10^13^ in their digestive tract [72] and are the dominant sources of bioaerosols in indoor environments [73–75]. Key factors that determine the composition of the indoor fungal and bacterial microbiome appear to be moisture, age of the home, and dog ownership [76]. Potential effects on health may come from fungi as a significant source of allergens and mycotoxins [77], whereby indoor fungal communities are dominated by species originating from outdoors [78]. Fungal and bacterial spores can infect animals, plants, and humans [79], are highly stable, and can survive dormant for years. Fungi such as *Cryptococcus* spp. can cause fatal disease in immunocompromised populations, such as AIDS patients and transplant recipients [80]. However, most microorganisms are benign and protect against harmful microbes, assist in the digestion, train the immune system, and lower the risk of autoimmune diseases [81]. High doses of pathogens are, however, a risk under poor sanitary conditions and exposure to droplets and aerosols from infected people with high titers of pathogens.

### 2.5. Healthcare Facilities and Transportation

Not surprisingly, the indoor air microbiota of hospitals contain a larger percentage of potential bacterial pathogens than do outdoor samples [82]. Indeed, many healthcare facilities are affected by the spread of SARS-CoV-2 and the resulting infection of healthcare workers and other patients. Microbiome studies of hospitals may help to reduce exposure to pathogens; for example, rooms with higher airflow and humidity were associated with fewer airborne human pathogens [82]. Thus, architectural design may help to reduce transmission of pathogens in healthcare facilities. Ventilation systems of trains and airplanes typically recycle cabin air which is passed through filters that do not efficiently remove viruses. During the SARS-CoV-2 pandemic, this has resulted in almost complete shutdown of long-distance traffic and public transport in many countries. Spread of the virus may only be prevented if all passengers are confirmed negative for SARS-CoV-2 infections via antibody testing or real-time viral tests indicating a virus-free status. Such tests are available for influenza virus; they provide rapid results but are often less reliable than laboratory tests. Yet, that may be the only fast solution for long-distance travel in trains or airplanes. Keeping a safety distance and masks can only help to contain the spread of SARS-CoV-2 to a certain extent.

### 2.6. Airborne Viruses and Phages

Much less is known about airborne viruses than about bacterial and fungal communities. The International Committee on Taxonomy of Viruses (ICTV) lists approximately 6,000 known viruses, of which about 1,500 can cause diseases [83]. Patients acutely infected with influenza virus can harbor up to 10^9^ virus particles per cubic centimeter in the blood stream and release approximately 10,000 aerosolized viruses by coughing or sneezing [75]. Indoors, influenza virus can reach concentrations of up to 2.6 × 10^5^ particles per cubic meter [68]. Even more infectious by airborne transmission is measles virus, which leads to almost 100% infections upon contact with an infected person [84]. Measles virus causes severe disease during childhood and can also be dangerous for adults, especially for pregnant women [84].

Noroviruses are relatively stable and can persist in the environment for several weeks [84]. As few as 18 to 1,000 norovirus particles can cause an infection [85]. Noroviruses account for about 50% of infectious diarrhea in humans. There are at least 33 genotypes and acquired immunity is short-lived and not cross-protective, so that a person may encounter several norovirus infections per year. Norovirus is usually not seriously harmful to healthy adults, but to young children and the elderly [84]. Closed environments such as cruise ships are commonly affected by norovirus outbreaks.

## 3. Coronaviruses

Coronaviruses are single-stranded positive-sense RNA viruses, with seven known to infect humans, including SARS-CoV-1, MERS-CoV, and SARS-CoV-2 [86]. The four others contribute to about 10–15% of the seasonal acute respiratory infections [87]. Other seasonal viral infections are caused by influenza A and B viruses, respiratory syncytial virus, and rhinoviruses. Respiratory viruses such as influenza or coronaviruses, including SARS-CoV-2, are transmitted by respiratory droplets (larger drops emitted by coughing, sneezing, or talking) and aerosols (particles smaller than 1 micron in diameter) when they reach susceptible mucosal surfaces of the eyes, nose, or mouth. Indirect contact through smear infections from contaminated surfaces may occur but the amount of viable viruses may be small. The transmission of respiratory viruses can be limited by wearing face masks, which reduce the spread of droplets and aerosols between people. Outdoors, the viruses are normally too sparse to pose a significant risk for infecting healthy people if a safety distance from other people is maintained. Even though droplets may travel a distance of about 30 cm before they sink, a safety distance for up to 2 meters has been proposed to contain the spread of SARS-CoV-2. Face masks covering the nose and mouth can reduce droplet-based viral infections, while only surgical masks may protect against viral aerosols.

Air pollution as reviewed here can cause lung damage. This is a prominent problem mainly in large cities and manifests itself as “Beijing cough,” a dry cough highly prevalent in large and polluted cities [19]. There is evidence that people exposed to severe air pollution are more susceptible to infection with the present SARS-CoV-2 pandemic virus and experience stronger symptoms, not only in large cities of China but also in other parts of the world [46–51]. Pollution, including PM and NO_2_, likely contributed to the spread of SARS-CoV-2 and severity of disease in Northern Italy where pollution is severe [46, 47, 49, 50]. In addition to air pollution, preexisting conditions such as overweight may contribute to disease severity, which may especially be relevant for the US, where close to 40% are clinically obese [58].

SARS coronaviruses have a history as pollutant through plumbing [88]. For example, SARS-CoV-1 spread through the plumbing of the Amoy Gardens Building in Hong Kong, which was not aerosol-tight and thereby allowed the virus to spread from the 7th floor of the 33-story building with contaminated sewage [88]. Also, in the Hotel Metropole in Hong Kong, twelve people were infected within 24 hours, causing a chain of infection of up to 4,000 people [89]. SARS coronaviruses are extremely contagious [90]. Strict regimens for infected people in Singapore successfully contained the SARS-CoV-1 outbreak. However, the virus even escaped twice from researchers working under high safety laboratory conditions [91].

Phages, the viruses of bacteria, are abundant on our planet, in the oceans, air, soil, and other environments [92]. They can integrate into bacterial genomes but can also replicate by lysing the bacteria. About 10–20% of bacteria in the oceans are lysed daily by phages [93]. It is not trivial to characterize phages in an environmental sample; they typically require purification, concentration, and PCR amplification steps prior to sequencing and taxonomic assignment [94, 95]. The identification of phages in human samples has recently been discussed in detail [95]. Phages were identified in the air of Seoul and may therefore spread through the air [32]. Yet, they are not known to pose a risk for human health.

### 3.1. Effects of Inhaled and Ingested Pollution

**Figure 3:**
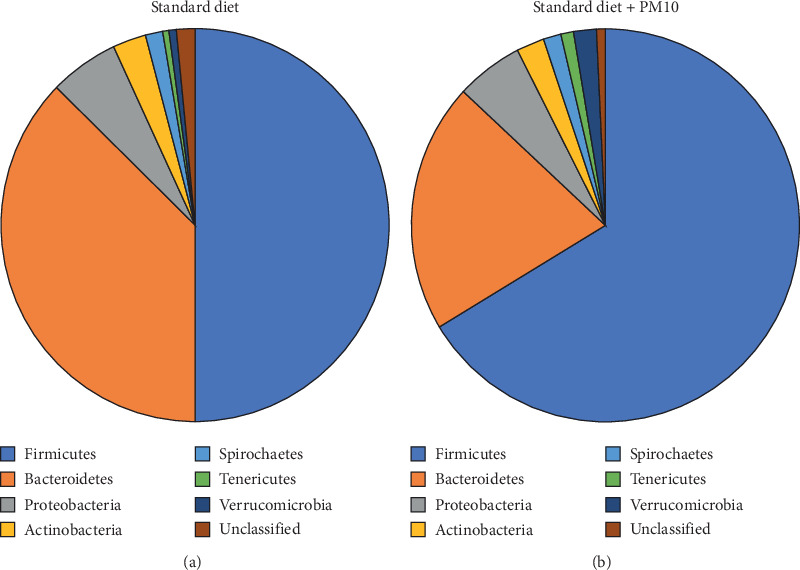
Changes in intestinal microbiota due to PM_10_ in a mouse model. IL-10 knockout mice, a model for inflammatory bowel disease, were fed with either standard mouse chow (left) or standard mouse chow supplemented with PM_10_ for 35 days [99]. Then, the bacterial composition in fecal samples of these mice was determined.

An important question is whether air pollution influences the composition of the host microbiota. The gastrointestinal tract harbors the highest number of microbes and may be indirectly affected by high concentrations of pollutant PM [96]. In humans, inhaled PM is rapidly cleared from the lungs and transported into the intestine where it may cause alterations in bacterial community compositions [97]. In a mouse model of inflammatory bowel disease (IBD), orally administered environmental PM_10_ at a concentration representing a dose that could occur during periods of high levels of air pollution has been shown to significantly affect the gut microbiota [98]. The proportion of Firmicutes was increased, while Bacteroidetes decreased and inflammatory responses and gut permeability were promoted (Figure 3) [98]. Epidemiological evidence suggests that air pollutants are also linked to an increased risk for IBD in humans [99]. It has been suggested that air pollution, in general, and PM, specifically, may promote gastrointestinal diseases in humans [86]. Recently, it has been shown that PM inhalation may alter the intestinal microbiota in humans [100]. As observed experimentally in mice, an increase in Bacteroidetes and a decrease in Firmicutes were observed, with health consequences yet to be determined.

In addition to IBD, exposure to air pollution has been linked to type 2 diabetes and obesity, possibly due to effects on the intestinal microbiota [101, 102]. Specific families of gut bacteria correlated with NO_*X*_ exposure; Bacteroidaceae (phylum Bacteroidetes) increased, while Coriobacteriaceae (phylum Actinobacteria) decreased [101]. These changes were associated with increased fasting glucose levels characteristic of developing type 2 diabetes. In addition, polycyclic aromatic hydrocarbons and other organic pollutants present in PM can be metabolized by gastrointestinal bacteria and thereby alter the composition of the microbiota [103].

Alterations in the lung microbiome have been linked to various diseases such as cystic fibrosis, COPD, and asthma [104]. For example, patients with asthma and COPD have increased relative abundances of Proteobacteria compared to healthy individuals. Interestingly, it has been shown that individuals exposed to higher levels of PM from household air pollution in Malawi showed alterations of their lung microbiome, including higher relative abundances of potentially pathogenic bacteria of the genera *Streptococcus* and *Neisseria* [105]. Moreover, domestic biomass fuel use was associated with the presence of an environmental bacterium, *Petrobacter,* which is normally not present in the lung [105].

In summary, there is evidence that environmental pollution can affect the composition of both the gastrointestinal and the lung microbiota, with potential negative effects on human health. Thus, air pollutants, without directly transporting microbes, can indirectly affect the body's inherent microbiota.

## 4. Discussion

We are only beginning to understand the composition of aerial microbiomes and their potential impact on human health. However, from the current data, the following trends emerge for bacterial, viral, and fungal communities, despite the varying methodologies employed by the different studies.

### 4.1. Airborne Bacteria

The bacterial communities of urban air microbiomes appear to be mainly composed of the phyla Proteobacteria, Actinobacteria, and Firmicutes (Figure 3), while less abundant populations such as Bacteroidetes and Cyanobacteria are more variable among samples [31, 32, 34–37]. This is reminiscent of bacterial and viral microbiota of the oceans and the human intestinal tract that are composed of abundant core members and less-abundant variable populations [55, 106]. Potential human pathogens are typically below the detection limit in air samples even from closed environments such as subway systems, which means that there is not likely a significant risk for infection [31, 32, 34–37].

### 4.2. Airborne Viruses

Likewise, ambient air appears to not contain significant amounts of known viral pathogens [33]. However, only a small fraction of all viruses found in the environment are known, which makes it difficult to estimate potential effects of the air virome on human health [33]. A major constituent of the airborne virome is bacteriophages that are not known to pose a risk for humans but may affect bacterial populations contributing to the spread virulence and antibiotic resistance genes [33].

### 4.3. Coronaviruses

SARS-CoV-2 is the cause of the current COVID-19 pandemic of 2019/2020, which has led to outbreaks of varying severities. High infection and death rates were observed, for example, in Wuhan city and other parts of China, Lombardy in Northern Italy, Northern Iran, New York City, USA, Manaus, Brazil, and Johannesburg, South Africa. In some cases, the severity of the outbreaks may have been linked to air pollution in conjunction with a high population density. Other risk factors may comprise overweight/obesity, chronic cough, lung diseases such as COPD, and infectious diseases such as tuberculosis and HIV/AIDS [44–50, 58, 107, 108]. SARS-CoV-2 most efficiently spreads through contact with infected people in indoor environments [109]. This has prompted restrictions of public transport and long-distance travel in many countries worldwide. Outdoors, virus-containing droplets or aerosols typically do not travel through air beyond the proposed safety distance of one to two meters in amounts sufficient to cause an infection.

### 4.4. Airborne Fungi

A major risk for human health is airborne fungi that can exacerbate diseases including allergies and asthma [31, 32, 35]. Studies on fungal air microbiomes may help to identify measures to reduce the abundance of fungal species linked to allergies, asthma, and other diseases in outdoor and indoor ambient air. For indoor environments, it has been shown that the abundance of specific components of the airborne microbiota can be altered by architectural design, humidity, and the degree of air flow [82]. Thus, hypoallergenic architectural design can be envisioned. There is evidence that fungal spores are particularly abundant in rural air [59]. Interestingly, exposure to indoor dust-borne *Alternaria* spp. was found to be linked to a reduced occurrence of asthma, whereas indoor airborne *Aspergillus fumigatus* and *Alternaria* spp. were positively correlated with asthma [110]. Thus, exposure to fungi may have both positive and negative consequences for human health, depending on the species and the type of exposure (e.g., dust-borne vs. air-borne). In general, however, it is difficult to compare current studies, as they relied on varying protocols. In the future, standardized methodologies will be helpful to allow for better comparisons between studies.

On a larger scale, there is evidence that the microbiome is globally connected [62] and that microbes may be transported over thousands of kilometers by dust and fine sand [63, 111] and through high altitudes up to the troposphere [38, 61]. Whether potential pathogens can cause an infection after exposure to high altitude and the associated radiation, however, remains to be shown. While a direct effect of microbes transported over long distances on human health, such as infections, is unlikely, a potential concern is the dissemination of virulence factors and antibiotic resistance genes [65].

Exposure to PM, even without attached microorganisms, has been shown to alter the intestinal microbiota and may be linked to diseases such as IBD [98–100] and type 2 diabetes [101, 102]. Whether exposure to specific airborne microbes also influences these diseases remains to be determined.

## Figures and Tables

**Table 1:** Recommendations and thresholds.

	WHO recommendations	EU thresholds	Chinese Ministry of Environmental Protection^*∗*^	United States EPA
PM_2.5_	10 *μ*g/m^3^ annual average	25 *μ*g/m^3^ annual average	35 *μ*g/m^3^ annual average	35 *μ*g/m^3^ averaged over 24 hours
	25 *μ*g/m^3^ 24-hour maximum		75 *μ*g/m^3^ daily average	

PM_10_	20 *μ*g/m^3^ annual average	40 *μ*g/m^3^ annual average	70 *μ*g/m^3^ annual average	150 *μ*g/m^3^ averaged over 24 hours
	50 *μ*g/m^3^ daily average	50 *μ*g/m^3^ daily average	150 *μ*g/m^3^ daily average	

NO_2_	40 *μ*g/m^3^ annual average	40 *μ*g/m^3^ annual average	40 *μ*g/m^3^ annual average	53 parts per billion (ppb) annual mean
	200 *μ*g/m^3^ 1-hour maximum	200 *μ*g/m^3^ 1-hour maximum	80 *μ*g/m^3^ daily average 200 *μ*g/m^3^ 1-hour average	

O_3_	100 *μ*g/m^3^ 8-hour maximum	120 *μ*g/m^3^ 8-hour average	160 *μ*g/m^3^ 8-hour average 200 *μ*g/m^3^ 1-hour average	0.070 parts per million (ppm) averaged over 8 hours

SO_2_	20 *μ*g/m^3^ daily average	125 *μ*g/m^3^ daily average	60 *μ*g/m^3^ annual average 150 *μ*g/m^3^ daily average	0.5 ppm averaged over 3 hours
	500 *μ*g/m^3^ 10-minute maximum	350 *μ*g/m^3^ 1-hour maximum	500 *μ*g/m^3^ 1-hour average	

Numbers in parentheses are the maximal numbers of allowed exceedances per year. ^*∗*^Values applying for urban areas are shown. Stricter standards are required for special regions such as national parks. Numbers are according to the WHO [1], the EU [2], the Chinese Ministry of Environmental Protection [5], and the United States Environmental Protection Agency (EPA, values retrieved from https://www.epa.gov/criteria-air-pollutants/naaqs-table).

**Table 2:** Common health effects caused by exposure to particulate air pollution [9–12].

Particle size	Short-term exposure	Long-term exposure
PM_10_	Allergies, asthma, bronchitis, COPD, coughing, eye irritations, hay fever, increased respiratory infections, and rhinitis	COPD
PM_2.5_	Asthma, cardiovascular disease, COPD, coronary heart disease, heart insufficiencies, hypertonia, and increased respiratory infections	Allergies, asthma, atherosclerosis, COPD, increased risk for cancer, and shortened life expectancy
PM_0.1_	Asthma and coronary heart disease	Unknown
